# Macroalgae—A Sustainable Source of Chemical Compounds with Biological Activities

**DOI:** 10.3390/nu12103085

**Published:** 2020-10-11

**Authors:** Elena-Suzana Biris-Dorhoi, Delia Michiu, Carmen R. Pop, Ancuta M. Rotar, Maria Tofana, Oana L. Pop, Sonia A. Socaci, Anca C. Farcas

**Affiliations:** 1 Department of Food Science, University of Agricultural Sciences and Veterinary Medicine Cluj-Napoca, 3-5 Calea Manastur, 400372 Cluj-Napoca, Romania; elena.biris@usamvcluj.ro (E.-S.B.-D.); carmen-rodica.pop@usamvcluj.ro (C.R.P.); anca.rotar@usamvcluj.ro (A.M.R.); maria.tofana@usamvcluj.ro (M.T.); oana.pop@usamvcluj.ro (O.L.P.); 2 Department of Food Technology, University of Agricultural Sciences and Veterinary Medicine Cluj-Napoca, 3-5 Calea Manastur, 400372 Cluj-Napoca, Romania; delia.michiu@usamvcluj.ro

**Keywords:** macroalgae, bioactive compounds, bioactivities, antimicrobial, antiproliferative activity, polysaccharides

## Abstract

Nowadays, one of the most important research directions that concerns the scientific world is to exploit the earth’s resources in a sustainable way. Considering the increasing interest in finding new sources of bioactive molecules and functional products, many research studies focused their interest on demonstrating the sustainability of exploiting marine macroalgal biomass as feedstock for wastewater treatment and natural fertilizer, conversion into green biofuels, active ingredients in pharmaceutical and nutraceutical products, or even for the production of functional ingredients and integration in the human food chain. The objective of the present paper was to provide an overview on the recent progress in the exploitation of different macroalgae species as a source of bioactive compounds, mainly emphasizing the latter published data regarding their potential bioactivities, health benefits, and industrial applications.

## 1. Introduction

Algae are part of a heterogeneous group of photosynthetic organisms. The division includes multicellular organisms, macroalgae or seaweed (reaching sizes of up to 60 m in length), and unicellular organisms, also known as microalgae (measuring from 1 mm to several cm). One way to classify macroalgae is on the basis of their pigmentation: (i) brown seaweed (*Phaeophyceae)*, (ii) red seaweed (*Rhodophyceae*), and (iii) green seaweed *(Chlorophyceae*) [1].

Algae are distributed in diverse and extreme environments. They are valuable due to their high content in compounds with different biological activities, including both complex organic compounds and primary and secondary metabolites. Worth mentioning, among them are phytopigments (xanthophylls and carotenoids), polyunsaturated fatty acids (PUFAs) comprising docosahexaenoic acid (DHA), phenolic compounds, tannins, peptides, lipids, enzymes, vitamins, carbohydrates, terpenoids, and others. Thus, algae are a viable and economical biomass source of valuable compounds with potential applications in the nutraceutical, pharmaceutical, chemical, food, and cosmetic industries due to their biologically active and regenerative properties [2,3,4,5,6].

In recent years, macroalgae have gained more and more interest owed to their various health promoting properties that can decrease the risks of many chronic diseases and even help to extend the lifespan [7,8]. Macroalgae can also be used for wastewater treatment or as a natural fertilizer in agriculture, therefore improving the quality of the products and minimizing the need for chemical fertilizers [9,10,11]. The potential of macroalgae as a source of renewable energy is also of considerable interest. These aquatic organisms have the ability to mitigate carbon dioxide emissions and nowadays are being used as feedstock to produce “clean” or so-called “third generation biofuels” [12].

**Figure 1:**
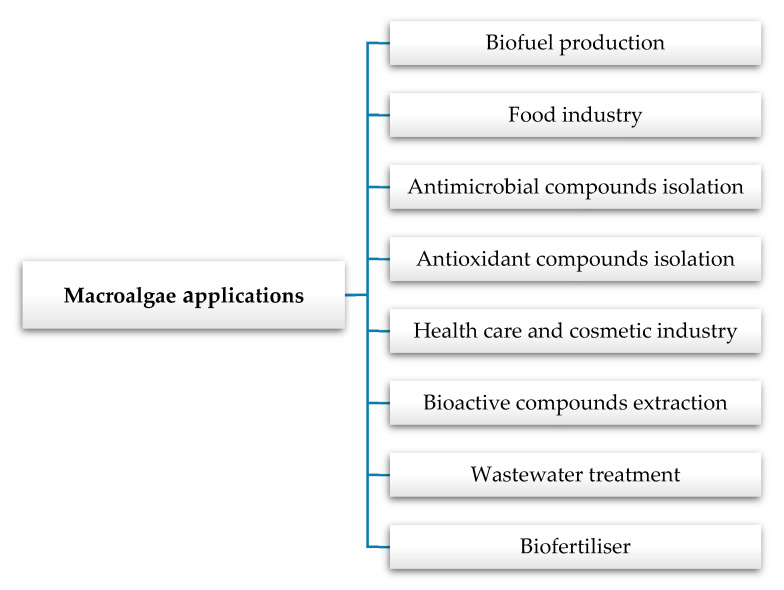
The main applications of macroalgae.

The most important applications of algae are synthetized in Figure 1.

This review focuses on the recent progress in exploitation of different macroalgae species as a source of bioactive compounds, mainly emphasizing the latter published data (between 2010 and 2020) regarding the health benefits, their bioactivities, and potential applications.

## 2. Algae Chemical Composition

The use of different marine macroalgae (seaweed) as sources of bioactive compounds had the advantage to exploit an under-utilized renewable natural resource. It was demonstrated that this biomass produced a broad spectrum of nutrient and bioactive secondary metabolites. The chemical composition of macroalgae varies considerably due to both environmental conditions (light intensity, growth habitat, seawater salinity, temperature) and genetic differences among species [2,13].

Macroalgae have a protein content that can range from 7 to 31% of dry weight and a lipid content ranging from 2 to 13% of dry weight [14]. A considerable amount of carbohydrate can also be found in macroalgae (up to 32–60% of dry weight).

Regarding the macroalgae content in micronutrients, they are a good source of vitamins, especially of the B-group representatives (i.e., B_1_, B_12_), as well as the lipophilic vitamins A and E (tocopherol) [13,15,16,17]. The richness in vitamin B_12_ propels the macroalgae-based products as dietary supplements for a vegan lifestyle, considered to be at risk for vitamin B_12_ deficiency [18]. Within the mineral composition, the most significant microelements present in the seaweeds are usually potassium, sodium, magnesium, and calcium, accounting for more than 97% of the total mineral content. Other microelements such as copper, iron, manganese, and zinc are found in small amounts (ranging from 0.001 to 0.094% of seaweeds’ dry weight) [19].

### 2.1. Protein and Amino Acid Composition

Proteins are a major class of compounds, essential for human nutrition. For food products, the amount of protein is considered a quality parameter, but of equal importance for human health is the protein quality (e.g., protein composition in amino acids, the ratio of essential amino acids, their digestibility, and bioavailability). It is well known that seaweeds can be used as a nutrient source, especially in developing countries. In this sense, macroalgae is considered a sustainable nutrient alternative source, mainly due to high-value proteins.

Nine of the 21 amino acids are considered essential for humans, namely: histidine, isoleucine, leucine, lysine, methionine, phenylalanine, threonine, tryptophan, and valine. Proteins of animal source have a chemical score of 1.0, meaning that animal proteins contain all the essential amino acids in a minimum proportion necessary for the human body. Instead, the chemical score for cereal proteins normally ranges from 0.4 to 0.6, while the one for algae proteins ranges from 0.75 to 1.0 indicating that the protein quality of algae is superior to most terrestrial plants [14]. Therefore, macroalgae are able to cover the human requirements for essential amino acids [13].

The protein content of marine algae differs according to species. Although the protein level is generally low in brown seaweeds (3–15% on dry weight basis (DW)), and moderate in green seaweeds (9–26% DW), in red seaweeds the content can reach 47% DW [20]. One gram of algae meal from algae with the highest protein levels (e.g., *Enteromorpha intestinalis*, *Palmaria palmata*, and *Vertebrata lanosa)* contains equal to or higher amounts of all of the essential amino acids compared to rice, corn, and wheat. In addition, the lysine content was reported to be three to nine times higher. The approximate amount of free amino acids can range from 2 to 14.5%, the lowest amount being reported in the green algae and highest in the red varieties [13]. If we consider nonessential amino acids, the green seaweed proteins contain high levels of glutamic and aspartic acids (that can have a concentration up to 26 and 32% of the total amino acids), but also alanine and glycine [20].

The seaweed varieties that have a high protein level can be used as ingredients in the manufacturing process of different foods. *Porphyra* species are known to be used in the famous sushi preparations. The same seaweeds are also processed into roasted products (such as yaki-nori) or they can be boiled in soy sauce (tsukudani-nori) [6]. For instance, species such as *Ulva pertusa*, *Enteromorpha* sp., and *Monostroma* sp. (protein levels of 26, 19, and 20% dw, respectively) are mixed together to create a food product called “aonori” (or green laver), a protein rich product very appreciated in Japan. In Europe and Canada, *Palmaria palmata* is often used as a food ingredient. Due to its high protein content (up to 35% dw), this specie of algae can be processed into dry flakes and used to obtain different functional products [21,22].

### 2.2. Lipid and Fatty Acid Composition

The lipid content is relatively low in macroalgae species, with values less than 5% w/dw. Variations in the quantity and in fatty acids profile can be attributed to both environmental (light intensity, seawater salinity, temperature) and genetic differences among species. In general, it has been observed that brown species have a higher lipid content compared to green varieties [23,24].

However, nearly half of lipids are polyunsaturated fatty acids such as eicosapentaenoic acid (EPA) and arachidonic acid (AA). Red and brown algae are rich in EPA and AA, while green seaweeds such as *Ulva pertusa* predominantly contain hexadecatetraenoic, oleic, and palmitic acids, and also significant levels of PUFAs, such as linoleic acid (18:2n-6) and 𝛼-linolenic acid (18:3n-3) [23,25]. Moreover, the ratio between ω-6 and ω-3 and the ratio between PUFAs and SFAs (saturated fatty acids) found in red and brown algae are more favorable for human health than those found in green algae [26].

Besides the fatty acids, the lipidic fraction of macroalgae contains glycolipids and phospholipids. Glycolipids are carbohydrates (mono- or oligosaccharide) that are linked to a lipid (through a glycosidic bound), being essential components of the cellular membrane. Several studies were conducted on different glycolipids from seaweed (e.g., monoglycosyl diacylglycerol subfraction from *Fucus distichus;* monogalactosyl diacylglycerols from *Sargassum horneri;* sulfoglycolipids from *Porphyra crispata)*, showing their anti-inflammatory and antiproliferative effects, respectively [27,28,29]. Regarding lipid extraction, Ramola et al. [30] and Margareta et al. [31] found that the most efficient solvent was a mixture of chloroform: methanol (2:1) with an efficiency of 14% compared to hexane (2:1) with 12.5% efficiency.

### 2.3. Carbohydrates

Carbohydrates, which include mono-, oligo- and polysaccharides, are considered an important and irreplaceable source of energy necessary to support different functions of the human body and its physical activity [32]. Of these, algal polysaccharides such as alginates, carrageenan, fucoidan, and laminarin, were found to exhibit a wide spectrum of biological activities, amongst which the antioxidant, antithrombotic, anti-inflammatory, and neuroprotective activities are the most studied [33]. Additionally, the nondigestible oligosaccharides can act as prebiotic agents gaining increased attention due to their positive influence on the gut flora [34,35,36]. The qualitative differences of the sugar backbone, the molecular weight, and also the sulfation degree vary the pharmacological effects of algal carbohydrates [37].

In general, edible seaweeds present a variable carbohydrate content. Reported to fresh weight, carbohydrates levels vary from 4.1/100 g wet weight in *Ulva* sp., to 13.1/100 g wet weight in species *Ascophyllum nodosum*, with species *Laminaria digitata* having a content of 9.9/100 g wet weight [3]. Furthermore, the *Undaria pinnatifida* species contains carbohydrates in a content of 9.14% [38]. Related to dry matter, the total carbohydrate concentrations in the seaweed species represent up to 76% of their dry weight. In this sense, species such as *U. pinnatifida* contains between 45 and 52% total carbohydrates. A high content of carbohydrates can be also found in *Saccharina japonica* brown algae (51.9% of dry weight), *Gracilaria chilensis* red algae (66.1% of dry weight), and *Ulva compresa* green algae (48.2% of dry weight) [17,39,40].

According to literature data, the sulfated polysaccharides represents one of the main constituents [41,42]. The highest contents are found in genera such as *Ascophyllum*, *Porphyra*, and *Palmaria*. Additionally, species *Kappaphycus alvarezii* and *Eucheuma spinosum* present a content of polysaccharides up to 56 and 40%, respectively [43]. Carrageenans are one of the major constituents of red seaweed cell walls representing 30 to 75% of the algal dry weight. Ulvans are the major constituents of green seaweeds cell walls representing 8 to 29% of the algal dry weight. Alginates and fucans are the major constituents of brown seaweeds cell walls representing between 17 and 45%, respectively 5 to 20% of the algal dry weight. Furthermore, brown seaweeds contain laminarin up to 35% of the algal dry weight [44]. Porphyran, a linear sulfated polysaccharide, was found to be one of the main components isolated from *Pyropia yezoensis* (edible red alga cultivated and consumed mainly in East and Southeast Asia) [45]. Considering the applicability in the food industry, seaweed polysaccharides such as agar, alginates, and carrageenan are the most important and economically feasible obtained products due to their rheological gelling and thickening properties [17].

### 2.4. Pigments

Macroalgae use light as energy source and pigments play a key role in gathering solar energy. These pigments absorb light from the visible spectrum [46,47]. Carotenoids are orange/red pigments that absorb light energy and then pass it on to chlorophyll, therefore playing a secondary role in photosynthesis [48]. Carotenoids supplement the light gathering potential of the algae. There is an alteration for both chlorophyll and carotenoid content in seaweeds depending on the ultraviolet radiation (UV) levels throughout the year. Both chlorophyll and carotenoid pigments possess antioxidant and chemo preventive properties [37,49]. The main algal pigments in commercial use at the present time comprise: beta-carotene, astaxanthin, lutein, phycocyanin, chlorophyll, and fucoxanthin.

#### 2.4.1. Carotenoids

Carotenoids are terpenoid pigments widely distributed that are divided into two main groups: carotenes (unsaturated hydrocarbons) and xanthophylls (carotenes’ oxygenated derivatives—of which in algae the most representatives are fucoxanthin, astaxanthin, lutein, and zeaxanthin) [50,51]. *β*-carotene is the major unsaturated hydrocarbon in brown and green seaweeds [37,52].

Carotenoids are a well-known as antioxidant agents [53]. Fucoxanthin exerts strong 2,2-diphenyl-1-picrylhydrazyl (DPPH) radical scavenging activity, most of it under anoxic conditions, being also recognized to exhibit anti-inflammatory properties. Its inhibitory activity against proinflammatory agents, such as nitric oxide (NO), tumor necrosis factor alpha (TNF-α), interleukin-1β, prostaglandin E2 (PGE2), and interleukin-6 (IL-6) was reported in [54]. A number of studies suggest that fucoxanthin is a promising and upcoming anticancer and antitumor agent and can suppress metastatic potential [55,56]. It also exhibited favorable levels of chemopreventive and/or chemotherapeutic activities against different human colon cancer cell lines, in combination with troglitazone being able to induce cell apoptosis via DNA fragmentation [57]. *U. pinnatifida* ethanolic extract, rich in fucoxanthin, was found to improve the plasma and lipid profile in high-fat diet mice. Aki et al. [58] investigated the effects of the seaweed carotenoids on unsaturated fatty acid metabolism in a hepatocyte culture (BRL-3A). The molecular mechanism revealed that fucoxanthin and its physiological metabolite, fucoxanthinol, caused alterations in fatty acid composition, leading to a decrease in EPA and the accumulation of docosahexaenoic acid.

#### 2.4.2. Chlorophylls

Chlorophylls are pigments which contain in their structure a central magnesium ion, playing a functional role in the algae photosynthesis process but also a protective role ensuring the algal tissue integrity against oxidative stress that may be excessive UV radiation [37]. Terrestrial plants and brown algae are dominated by chl a, while chl b is mainly related to green algae. Additionally, brown algae are considered the main source of chl c, while chl d is specific to red algae [59]. Chlorophyll is known to be converted into pheophytin, pyropheophytin, and pheo-phorbide in processed vegetable food and following ingestion by humans. These derivates show antioxidant and antimutagenic effect and may play a significant role in cancer prevention [17]. Beside the biological activities and health effects of the different chlorophyll catabolites, the seaweeds can also be considered as an alternative to replace the synthetic pigments used in the food industry.

### 2.5. Phenolic Compounds

Among the current interests of the scientific community is to find a sustainable source of bioactive molecules in order to reduce the use of synthetic compounds. In this sense, macroalgae phenolic compounds have gained particular attention due to their specific bioactivities and health-promoting benefits, including antioxidant, antiproliferative, antimicrobial, antiallergic, antidiabetic, and neuroprotective properties [60,61,62,63]. Similar to terrestrial plants, these secondary metabolites are essential to the normal growth and development of macroalgae, supporting the natural defense system against various disturbing factors such as diseases, injuries, and environmental aggression [64]. Structurally, phenolic compounds from terrestrial plants are derived from gallic and ellagic acid, while the algal compounds are derived from polymerised phloroglucinol units (1,3,5-trihydroxybenzene) [17].

The phenolic compounds present in macroalgae vary from simple molecules, such as phenolic and cinnamic acids or flavonoids, to the more complex phlorotannin polymeric structures, their concentration being closely dependent on a number of intrinsic and extrinsic factors, such as species, seasonal variations, and environmental conditions [64]. Of all the seaweed phenolic metabolites, the main attention has been focused on phlorotannins (phloroglucinol, eckol, 7-phloroeckol, 6,6-bieckol, phlorofucofuroeckol A, fucodiphloroethol), identified in considerable quantities in brown *Ecklonia* species [40]. Other compounds such as hydroxybenzoic acid derivatives (gallic, p-hydroxybenzoic, vanillic, and syringic acids), hydroxycinnamic acids (caffeic, ferulic, sinapic, and p-coumaric acids), flavonoids (epicatechin, epigallocatechin, rutin, quercitrin, hesperidin, myricetin, and kaempferol), and bromophenols were identified in variable concentrations in all green, red, and brown species [17,40,64].

## 3. Algae Applications in the Food Industry

Algae species have been used as plain food since ancient times. In Asia and in the East, the tradition of eating algae is a long-standing one, while in the Western countries, the interest in consuming algae-based products is quite recent but gaining increasing terrain [65]. Algae have manifold uses in different industry fields as a result of their rich chemical composition and content of bioactive substances. Moreover, their gelling, thickening, and stabilizing properties have driven the isolation and development of products such as agar, alginate, and carrageenan [66,67]. Due to these properties, algae have a main use in the food industry as hydrocolloids or as functional ingredients in different fish and meat products (steaks, frankfurters, or sausages), milk-based and fermented products [68,69,70], or cereal-based products (flour, pasta, bread, and biscuits) [4,21]. Moreover, these algae-based hydrocolloids are of utmost importance for food industry innovative fields such as molecular gastronomy.

In the dairy industry, algae were added in order to improve the nutritional value of cheese and other milk-based products [68]. *Laminaria* was added to smoked cheese, yoghurt, and milk deserts, giving them not only improved nutritional properties but also good sensory characteristics. *Laminaria saccharina* algae from the North Sea can be also introduced into cottage cheese or fresh cheese composition in order to improve their iodine content [69]. In addition to their nutritional properties, algae have been shown to have the ability to increase product stability during shelf-life due to the presence of compounds with antibacterial and antioxidant potential. In this sense, it was demonstrated that when algae Wakame (*U. pinnatifida*) and Kombu (*Laminaria japonica*) were added into the cheese composition, the product quality was maintained for a longer storage period [70]. In the meat industry, algae were added in the diet of lambs and chickens in order to improve the content of DHA, EPA [71,72], and antioxidants [73,74].

Recent studies on the bioactivity of some common species, such as *H. elongata* and *U. pinnatifida*, recommend their use in the composition of functional foods, due to the high content of antioxidants and the potential to alleviate the metabolic syndrome [75]. A wide range of studies reported the high potential of using algae as a source of prebiotics [76]. Wang et al. [77] proved that rats which had 2.5% alginate supplementation exhibited an increase in *Bifidobacterium* and *Lactobacillus*. A dietary supplementation of 1% laminarin was proved to result in an increase in *Bifidobacterium* number in rats [78]. *U. pinnatifida* and *Porphyra ternera* extracts fed to rats resulted in lower bacterial enzyme activity in the cecum, and also, the enzymatic activities that were reduced are implicated in the conversion of procarcinogens to carcinogens, therefore implying a possible link between seaweed extract intake and the reduced risk of colon cancer [79].

The most use species of algae in the food industry are summarized in Table 1 below.

## 4. Health Effects

**Figure 2:**
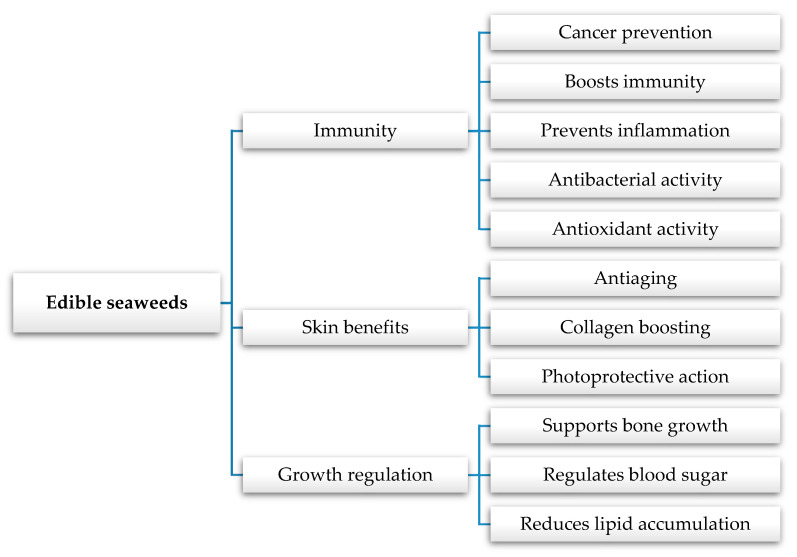
Health effects of macroalgae on human health and wellbeing.

Seaweeds contain a large variety of bioactive compounds that may be involved in the prevention and treatment of many diseases. They have several mechanisms for disease prevention and/or treatment. In this regard, some epidemiological, clinical, and meta-analysis studies associate the lower incidence of different chronic diseases, such as cancer, cardiovascular deficiency, diabetes, Parkinson disease, obesity related disorders, and metabolic syndrome, with a diet profile that includes seaweed consumption [49,90,91,92,93]. The main benefits on human health are presented in Figure 2.

### 4.1. Blood Pressure, Sugar, and Fat Reduction

Seaweeds are known to be rich in linolenic acid and its derivatives. These compounds can reduce blood viscosity and also smooth the interaction between blood vessels and vasoconstrictor substances. It was shown that when linolenic acid concentration increased by 1%, the blood pressure can diminish by 5 mmHg [90,94,95,96]. In this sense, Ryan et al. [86] analyzed the possible reduction in blood pressure using DHA algae oil and found that blood pressure reduction and heart rate were significantly reduced.

Alginate was shown to reduce blood sugar level, with sodium alginate supplementation of patients with diabetes type II leading to a decrease in the blood peak glucose level [96,97,98,99]. Porphyran and peptides were also proved to reduce blood sugar and blood pressure in rats and rabbits (*Porphyra yezoensis* in 1.6 g/L and 0.47 mg/mL) [100,101]. Fucoidan can reduce blood fat and sugar levels by disrupting fat absorption. Fucoidan is known to improve endoplasmic reticulum stress-reduced insulin sensitivity through adenosine monophosphate-activated protein kinase activation, and it can restore lipid homeostasis in mice with type II diabetes [102,103]. Linolenic acid aids the transformation of low-density lipoprotein (LDL) cholesterol to high-density lipoprotein (HDL) cholesterol and, therefore, can regulate fat metabolism [90,96,104].

### 4.2. Anticoagulant and Antithrombotic Properties

According to WHO, cardiovascular and cerebrovascular diseases have become the main cause of population mortality. Sulfated polysaccharides extracted from algae possess anticoagulant and antithrombotic properties [90]. In this regard, Ustyuzhanina et al. [105] showed that chemical transformation of branched xylofucans isolated from the brown algae *Punctaria plantaginea* into highly sulfated linear fucans effectively inhibited clot formation, having similar antithrombotic and anticoagulant effects to that of the heparinoid Clexane (enoxaparin) and the native fucoidan from *S. latissima*. *E. cava* was proved to be a great source of bioactive marine polyphenols, with antihyperglycaemic, antihyperlipidaemic, anti-inflammatory, and antioxidant effects, supported by evidence from in vitro studies as well as from those from human and animal trials already completed [60].

### 4.3. Antiaging, Antidepression, and Antifatigue Properties

A number of different physical and physiological factors are relevant when it comes to aging. The healthy function of the kidney and spleen plays an important role in human health. Seaweeds and seaweed-derived bioactive substances regulate the nervous system function, repairing DNA, promoting immunity, removing free radicals, regulating endocrine function, promoting healthy metabolism, and enhancing the kidney and spleen function [106]. *Fucus vesiculosus* aqueous extract increases the expression of integrin molecules. Topical application of the extract had a positive effect on the thickness and mechanical properties of human skin [90,96,106,107]. Polysaccharides have a large number of applications in the cosmetic industry. They act as rheology modifiers, suspending agents or wound-healing agents [108]. Carotenoids are powerful antioxidants possessing anti-inflammatory and antiaging properties. Several studies reported that astaxanthin, a xanthophyll carotenoid found also in macroalgae, can lower the oxidative stress protecting the mitochondria from the cumulative reactive oxygen species damage. Furthermore, astaxanthin was show to exhibit neuroprotective effects suggesting its possible use in the therapeutic treatment or prevention of neurodegenerative diseases such as Alzheimer’s or Parkinson’s disease [109,110].

Miyake et al. [111] performed a study on seaweed consumption and depressive symptoms during pregnancy concluding that a rich seaweed diet can be associated with a lower prevalence of depressive symptoms during pregnancy. Seaweed polysaccharides also possess antifatigue properties [112]. Higher hemoglobin, more oxyhemoglobin dissociation, and enhanced release of oxygen are responsible for the antifatigue property.

### 4.4. Antimicrobial and Antioxidant Potential

The antimicrobial compounds in algae are from several chemical classes, their level varying during algal growth and during seasons. In this sense, it was demonstrated that the *Polysiphonia* type produces antibiotic compounds constantly throughout the year, the *Laminaria* type has the maximum production during the winter, the *Dictyota* type during the summer, while *Codium* type has the best efficiency during the spring [113].

Extracts obtained with different solvents from a wide range of algae species, including *Ulva fasciata, Bryopsis plumosa, Chaetomorpha antennina, Acrosiphonia orientalis, Sargassum wightii, Grateloupia filicina, Hypnea pannosa, Gracilaria corticate Portieria hornemannii, Cheilosporum spectabile, Centroceras clavulatum, Chnoospora bicanaliculata, and Padina tetrastromatica*, were tested for their antimicrobial activity against *E. coli*, *S. aureus*, and *S. pyogenes*. From the tested solvents, the mixture between methanol and toluene (3:1 v/v) had the highest efficiency in extracting the compounds with antimicrobial potential from fresh biomass [114,115]. In another study, Tuney et al. [116] used methanol, acetone, diethyl ether, and ethanol to extract the bioactive compounds from 11 seaweed species. Diethyl ether extracts of fresh *C. mediterranea*, *E. linza*, *U. rigida*, *G. gracilis*, and *E. siliculosus* exerted high antimicrobial effects (10–15-mm halo) against several organisms (including *Enterococcus faecalis, Staphylococcus aureus, Pseudomonas aeruginosa*, and *Escherichia coli*). Instead, Bhuyar et al. [117] tested the ethanolic extract of the red alga *Kappaphycus alvarezii* against *Bacillus cereus*, the results indicating an inhibition zone with less than 10 mm of diameter.

The *Phylum Rhodophyta* (red algae) is recognized as one of the oldest groups of algae, characterized by the presence of phycoerythrin (a red protein-pigment complex), carrageenan (a sulfated polysaccharide), and phlorotannins. All of these compounds having strong antimicrobial activity. Another red alga extracts, *Symphyocladia latiuscula*, were proved to exhibit antimicrobial activity against a broad spectrum of microorganisms, the strongest antimicrobial effect being observed against *Vibrio mimicus* (50 μg/mL) and *Vibrio vulnificus* (50 μg/mL) [113,118].

Species such as *Laminaria saccharina*, *Laminaria digitata*, *Himanthalia elongata*, *Palmaria palmata*, and *Enteromorpha spirulina* are recognized as edible algae. Among these, *H. elongata* contains considerable amount of phenolics, tannins, and flavonoids. These antioxidant compounds that have a significant DPPH scavenging activity (50% inhibition (EC_50_) level at 0.125 μg/mL extract) can promote *H. elongata* as a natural alternative for food preservation. Moreover, the *H. elongata* methanolic extract at a concentration of 6% inhibited the growth of food spoilage (*Pseudomonas aeruginosa* and *Enterococcus faecalis*) and food pathogenic microorganisms (*Listeria monocytogenes* and *Salmonella abony*). Lower concentrations of the same brown seaweed extract (3%) extended the lag phase and decreased the exponential growth rate and final population densities of microorganisms in the culture [61,119].

The antimicrobial activity of other bioactive compounds extracted from marine algae was assessed against various microorganisms such as *Staphylococcus aureus*, *Salmonella choleraesuis*, *Mycobacterium smegmatis*, *Candida albicans*, and *Escherichia coli*. From the isolated compounds, three of them (namely cycloeudesmol (10–50 µg/mL), laurinterol (1–5 µg/mL), and debromolaurinterol (10–50 µg/mL)) exhibited antimicrobial activity at concentrations close to that of streptomycin (complete inhibition after 48 h) [120,121].

Studies of Al-Saif et al. [122] revealed the high antimicrobial potential of several algae strains (*Ulva reticulate*, *Caulerpa occidentalis*, *Cladophora socialis*, *Dictyota ciliolate*, and *Gracilaria dendroides*) against *Escherichia coli* (ATCC 25322), *Enterococcus faecalis* (ATCC 29212), *Pseudomonas aeruginosa* (ATCC 27853), and *Staphylococcus aureus* (ATCC 29213). The chloroform extract of *Gracilaria dendroides* had the highest antimicrobial activity against *E. coli* (32.6 mm inhibition zone). Wahidi et al. [123] tested the antimicrobial activity of extracts of macroalgae from Marrocan Atlantic coast against Gram-positive (*Bacillus subtilis* and *Staphylococcus aureus*) and Gram-negative (*Escherichia coli* and *Pseudomonas aeruginosa*) bacteria. Their results showed that the ethanolic extract of *Cystoseira brachycarpa* (500 μg/disc) had the highest inhibition diameter (>20 mm) for all tested bacteria, similar to that of control (rifampicine 30 μg).

In general, the microbial species on which the algae extracts have the strongest inhibitory activity are *Staphylococcus aureus* [9], *Escherichia coli* [124], *Salmonella* spp [62,125,126], *Bacillus cereus* [127,128], and *Listeria monocytogenes* [129,130]. For example, 100% ethanolic extracts of *Pithophora oedogonium* and *Botrydiopsis arhiza*, at concentrations of 2, 4, 6, and 8 mg/mL, were investigated for their antimicrobial activity against *Salmonella* and *Staphylococcus* sp. While *B. arhiza* extracts showed no inhibition capacity, the *P. oedogonium* extract (4 mg/mL) inhibits the growth of the above-mentioned strains [126]. Jang and Lee [128] evaluated the antibacterial potential of 51 Korean domestic algae methanolic extracts against foodborne pathogens, such as *B. cereus*, *S. aureus*, and *L. monocytogenes*. From the tested extracts, microorganisms were specifically sensitive to *Laurencia okamurae* Yamada and *Dictyopteris undulata* Holmes extracts which exerted antibacterial potential comparable with that of streptomycin [128]. *C. linum* methanolic extract at a concentration of 500 μg/mL was most effective against *B. cereus*, with a 27 mm inhibition zone, comparable with that of the standard antibiotic (chloramphenicol, 100 µg/mL). The high antimicrobial activity of the *C. linum* methanolic extract may be associated with its significant phenolic content (672.3 mg/g gallic acid equivalent), and high scavenging activity (IC_50_ 9.8 μg/mL) [62].

In the effort of finding new natural antimicrobials, the algae represent a rich source of bioactive compounds with manifold activities. In this direction, several studies were conducted assessing different fractions of methanolic or ethanolic seaweed extracts. For example, the ethyl acetate soluble fraction of *E. cava* methanolic extract exhibits high antibacterial activity against *L. monocytogenes* having an minimum inhibitory concentration (MIC) value of 256 µg/mL and an minimum bactericidal concentration (MBC) value of 512 µg/mL. Instead, the chloroform fraction of the ethanolic extract of *Myagropsis myagroides* was even more efficient in inhibiting the *L. monocytogenes* growth, with an MIC value of 63 μg/mL [129,130].

One worthy nutritional property of algae is linked to their high content of polyphenols, flavonoids, and carotenoids [131]. The major phenolic compounds isolated from the marine algae included anthraquinones, coumarins, and flavonoids, with rutin, quercetin, and kaempferol flavonoids being identified in all the algal species. According to Al-Saif et al. [122], the highest concentration of these three flavonoids was found in alga *Gracilaria dendroides* (rutin, 10.5 mg/kg; quercetin 7.5 mg/kg; kaempferol 15.2 mg/kg). These compounds were proved to be the most effective flavonoids in inhibiting bacterial growth (*E. coli*, *P. aeruginosa, S. aureus*, *E. faecalis*). The eckol (phlorotannin compound) isolated from the ethyl acetate extracts of *E. cava* species showed potential antimicrobial activity against methicillin resistant *S. aureus,* the MIC values ranging from 125 to 250 μg/mL [132]. In the case of phlorotanins isolated from *E. bicyclis*, namely eckol, dieckol, dioxinodehydroeckol, fucofuroeckol-A, 7-phloroeckol, and phlorofucofuroeckol-A, the MIC values for the inhibition of *S. aureus* and methicillin-resistant *S. aureus* ranged between 32 and 64 μg/mL [133]. The antioxidant activity of three representative Black Sea macroalgae, *Ulva lactuca* (green algae), *Cystoseira barbata* (brown algae), and *Ceramium rubrum* (red algae), was assessed according to the antioxidative capacity in lipid soluble substances procedure (ACL method). Of these, *C. barbata* showed the highest antioxidant activity (141.5 Trolox equivalent units, nmols/g dry weight) [134].

### 4.5. Antiallergic Effect

The worldwide trend is to use natural substances to cure allergies, and this has led to an increased interest in algal bioactive compounds, particularly in seaweed phenols. From the phenolic compounds, curcumin, epigallocatechin gallate, flavonoids, and quercetin were proved to have a significant antiallergic activity [20,21]. Additionally, fucoidan extracted from *U. pinnatifida* was proved to reduce the chemical and immunological responses in an animal model [21,135,136].

The porphyran, a sulfate polysaccharide isolated from *Porphyra tenera* and *Porphyra yezoensis*, is also known to possess antiallergic properties. The oral administration of porphyran (obtained from dried nori, 2% in drinking water) to mice with ear edema suppressed the evolution of the disease [137]. Aside from the antiallergic potential, the porphyran was also found to exert anti-inflammatory activity, their reactive oxygen species scavenging potential being considered the main mechanism responsible for this action [21]. Phlorotannins from *E. arborea* have been used since ancient times as folk medicine due to their antiallergic properties as reported by literature data [138]. Phlorotannins, carotenoids, polysaccharides, PUFAs, and phycocyanins were all found to exhibit antiallergic properties [139].

### 4.6. Anticancer Properties

For several decades, macroalgae have been promoted for their potential role in preventing cancer occurrence, tumor progression, and even health recovery after radio- or chemotherapy treatments [78,136]. Iodine may also exert an anticancer effect, due to the ability to cause apoptosis in cancer cells. The same property can be attributed to the omega-3 fatty acids such as stearidonic acid and hexadecatetraenoic acid found in edible marine algae such as *Undaria* and *Ulva* up to 40% of total fatty acids [140].

Alginate, laminaran, fucoidan, and many other seaweed polysaccharides were proven to have antitumor activities. A high amount of polysaccharide (~65% of polysaccharide in total dry weight) can be found in the many seaweeds such as *Ulva, Ascophyllum, Porphyra*, and *Palmaria* [141]. Alginate is able to clean up the intestinal tract, therefore improving immunity and intestinal tract health levels, reducing the risk of cancer. Laminaran and fucoidan are able to induce apoptosis in order to prevent cancer, but some unidentified seaweed polysaccharides can also exhibit direct or indirect antitumor effects. *Sargassum latifolium* inhibited cytochrome P450 1A and glutathione S-transferases and reduced 1301 cell viability, inducing apoptosis [142]. *Ulva fasciata* extract inhibits the growth of tumor cells in human colon cancer by 50% at a concentration of 200 µg/mL [143]. *Gracilariopsis lemaneiformis* was shown to have antitumor activity by inducing apoptosis in several cancer cell lines (e.g., human lung cancer cell line A549, the gastric cancer cell line MKN28, and the mouse melanoma cell line B16) attributed to neutral polysaccharide with a linear structure of repeated disaccharide agarobiose units [144]. Furthermore, the extracts of *Hydroclathrus clathratus* and its purified polysaccharide fractions were able to suppress the ascitic Sarcoma 180 tumor growth and prolonged the life span of the tumor-bearing mice with 30–40%, while presenting low toxicity to the normal cells [145].

Fucoidans extracted from *Dictyota ciliolata*, *Padina sanctae-crucis*, and *Sargassum fluitans* brown algae were reported to possess a high antioxidant activity being able to protect HepG2 cells from oxidative stress. Moreover, at a concentration of 2mg/mL, none of the fucoidan extracts had cytotoxic effects [146]. The isolated fucoidan from *Sargassum polycystum* exhibited potent antioxidant, anticancer, and antiproliferative properties against human breast cancer cell line MCF 7 at 150 g/mL and an IC50 of 50 g/mL [147]. Fucoidans from *Fucus evanescens* at a concentration of 800 μg/mL were reported to have anticancer properties by inhibiting the proliferation of melanoma SK-MEL-28 cell [148]. These findings underline once more that algae fucoidans are worth considering for the development of functional food, supplements, or drugs that can be used in the prevention of oxidative stress induced diseases.

The administration of extracts of the red alga *Eucheuma cottonii* to rats significantly improved the oxidative state of cells, and contributed to the tumor suppression response against MB-MDA-431 cell lines [149].

Meroditerpenoids are reported to be present mostly in brown algae. Metabolites such as fallahydroquinone, fallaquinone, fallachromenoic acid, sargaquinone, sargaquinoic acid, sargahydroquinoic acid, and sargachromenol were identified from the *Sargassum fallax* brown algae and reported to possess lower to moderate antitumor activity against a P388 Murine Leukaemia cell line [150].

### 4.7. Anti-Inflammatory Property

Inflammation is one of the most complex medical problems. It can be initiated by several factors, namely environmental pollution, chemical intoxication, and bacterial infection, which lead to injury or death of cells. Approximately 20% of all human cancers are caused due to chronic inflammation [151]. Several studies on the anti-inflammatory effect of different species of algae are listed in Table 2 below.

### 4.8. Antifungal Effects

The antifungal effects of macroalgae were also assessed by many studies. Species such as *E. cava* were found to have potential as a novel antifungal agent against *T. rubrum* at an MIC of dieckol of 200 μM [168], while *Halimeda tuna* methanolic extracts were very effective against *Aspergillus niger*, *Aspergillus flavus*, *Aspergillus alternaria*, *Candida albicans*, and *Epidermophyton floccosum* [7]. Studies of Ertürk et al. [169] evaluated the antifungal activity of *Enteromorpha linza* and *Padina pavonica*, and it was found to be stronger than the standard antifungal activity (100 units of nystatin) against *Aspergillus niger* and *Candida albicans*. Instead, the acetone extract of *T. conoides* exerted a mild antifungal capability against *Aspergillus niger*, with an inhibition zone diameter close to 3 mm [170]. In a study performed on 45 chronic asthmatic patients with acute respiratory distress, Mickymaray et al. [171] observed the highest antifungal activity against *C. albicans* in the case of *L. paniculata*, followed by *U. prolifera*, *Cladophoropsis* sp., *A. specifera*, and *Tydemania* sp. ethanolic fraction extracts. The minimum fungicidal concentration and MIC values of the above algal ethanolic fractions ranged between 125 and 1000 µg/mL and 125 and 500 µg/mL, respectively [171].

Like most bioactivities of algae, the antifungal activity of seaweed extracts can be related to the presence of phenolic compounds, polyunsaturated fatty acids, and various terpenoids [172,173]. For example, bromophenols were successfully isolated from the red alga *Odonthalia corymbifera*, and others obtained by chemical transformation of bis(hydroxyphenyl)methanes with bromine were tested for antimicrobial and antifungal activities by Oh et al. [174]. Among the natural bromophenols, 2,2′,3,3′-tetrabromo-4,4′,5,5′-tetrahydroxydiphenylmethane isolated from *Odonthalia corymbifera* showed good antifungal activity against *C. albicans*, *A. fumigatus*, *T. rubrum*, and *T. mentagrophytes* while two of the synthesized compounds exhibited effective antibacterial activity against *S. aureus*, *B. subtilis*, *M. luteus*, *P. vulgaris*, and *S. typhimurium* [174].

### 4.9. Antiviral Effects

Although the research regarding the antiviral potential of algae against food-borne viruses is gaining interest in recent years, currently, the available data are still scarce. The main compounds from algae that have been proved to have antiviral potential are sulphated polysaccharides, including fucoidan, sulphoglycolipids, carrageenan, and sesquiterpene hydroquinone. Marine-derived polysaccharides and their lower molecular weight oligosaccharide derivatives have been shown to possess a variety of antiviral activities and also exert antioxidant and antimicrobial effects. In general, algal polysaccharides can suppress the DNA replication and inhibit the host cell colonization by the virus. For example, the antiviral potential of polysaccharides from brown seaweeds revealed a significant inhibiting activity against hepatitis B virus (HBV) DNA polymerase, therefore affecting its replication [175]. The antiviral activity of these polysaccharides is exerted through suppression of virus adhesion to the host cells (*U. pinnatifida*, *Cystoseira indica*, *Ascophylum nodosum*) [176]. Fucoindan extract from *Cladosiphon okamurans* was used to inhibit New Castle Disease Virus in vitro in early stages of viral infection (0–60 min post-infection), the compound displaying high selectivity index (IS50 > 2000) for inhibiting syncytia formation [177]. *Grateloupia indica*, *Scinaia hatei*, *Gracilaria* corticata, *Stoechospermum marginatum*, *Cystoseira indica*, and *Caulerpa racemosa* sulfated polysaccharide extracts were screened for antiviral activity against the four serotypes of dengue virus (DENV). DENV-2 was the most susceptible serotype to all polysulfates, with inhibitory concentration 50% values in the range 0.12–20 μg/mL [178]. Krylova et al. [179] found that modified and native fucoidans from marine macroalgae *Fucus evanescens* presented antiviral properties against herpes virus or human immunodeficiency virus. Eom et al. [180] studied the antiviral activity of phlorotannin from *Eisenia bicyclis.* The results showed a strong antiviral potential against norovirus (murine norovirus, MNV) with EC_50_ of 0.9 μM [180]. Serkedjieva [181] analyzed the influence of *Ceramium rubrum* water extract on the reproduction of a range of influenza viruses in vitro and in ovo. The results showed that the virus-inhibitory effect was selective, dose-dependent, and strain-specific. At a concentration over 0.5 mg/mL, the extract also inhibited the reproduction of herpes simplex virus (HSV) type 1 with MIC_90_ of 1.4 mg/mL.

However, there is still the need for more research to comprehensively understand the antiviral action mechanisms of algae compounds and to benefit from their use as functional ingredients in pharmaceutical and food industries [124].

## 5. Conclusions and Future Trends

Nowadays, macroalgae are gaining more interest due to their demonstrated health promoting properties. They can be seen as a valuable source of bioactive compounds that can sustain the human health, preventing or reducing the convalescence period for various diseases, due to their antioxidant, anti-inflammatory, antiproliferative, antiviral, and antibacterial activities.

Although algae have been extensively analyzed regarding their content in biologically active compounds, the potential beneficial and toxicological effects on the human body are still of major interest. The identification of compounds directly responsible for the antimicrobial, antiviral, and anticancer activities of algae is still a relatively incipient domain that must be elucidated. Furthermore, the research must be oriented on their use as a substitute for antibiotics through a viable and sustainable approach, this strategy representing progress in solving the major emerging problems related to antibiotic resistance.

A future valorization strategy can be sustained trough an integrated biorefinery concept developed based on cost-effective and environmentally-friendly extraction methods. In this context, more research is needed to evaluate nutritional properties and mechanisms underlying the health benefits of a wide variety of macroalgal products.

## Figures and Tables

**Table 1:** The most used species of algae in food industry and their general characteristics.

*Laminaria digitata*	Dark brown, to 2 m in length; presents a claw-like holdfast, a smooth, flexible stipe, and also a laminate blade to 1.5 m long split into finger-like segments [80]
	The species is rich in alginates, mannitol, and amino acids. [20,81]
	Very rich in iodine; therefore, this seaweed promotes slimming and has antiseptic properties [82]
*Dictyota dichotoma*	Thallus is flat and leaf-like, up to 30 mm long and 5–30 mm broad. The fronds are thin and translucent; colors differ from olive to golden-brown [80]
	Produces large amounts of heterogeneous polysaccharides when submitted to the extraction procedures used to obtain fucoidans [83]
*Undaria pinnatifida*	Thallus fixed by a ramified holdfast [84]
	Rich source of eicosapentaenoic acid, an omega-3 fatty acid, and presents high levels of sodium, calcium, iodine, thiamine, and niacin [16,38]
*Enteromorpha linza*	Can be distinguished by its smooth thallus, most with a ruffled margin [85]
	Rich in essential amino acids, fatty acids, vitamins, dietary fiber, and resistant protein [2,3,86]
*Ecklonia cava*	A perennial brown alga and exists mainly in subtidal areas off the coast of Japan and Korea [87]
	Eckol isolated from *E. cava* attenuates oxidative stress-induced cell damage in lung fibroblast cells [88]
	Exhibits radical scavenging activity, but also antiplasmin inhibiting activity, antimutagenic activity, bactericidal activity, human immunodeficiency virus type 1 (HIV-1) reverse transcriptase, and protease inhibition [89]

**Table 2:** The anti-inflammatory effect of different species of algae.

Algae Species	Active Extract/Compound	Biological Activity	Reference
***Phorphyra dentate***	Methanolic extracts	Anti-inflammatory effect in lipopolysaccharide (LPS) induced mouse RAW 264.7 macrophages cell line.	[152]
***Caulerpa mexicana***	Methanolic extracts	Decrease the xylene-induced ear edema and reduce cell migration to different sites.	[153]
***Myagropsis myagroides***	Fucoxanthin	LPS-stimulated RAW 264.7 macrophages.	[154]
***Ulva reticulate***	Methanolic extracts	Carrageenan-induced hind paw edema in rats and peritonitis in acute and chronic inflammatory models.	[155]
***Laminaria saccharina***	Sulfated polysaccharides	Inhibits leukocyte recruitment in rat and the neutrophil adhesion to platelets.	[156]
***Porphyra dioica*** ***Palmaria palmate*** ***Chondrus crispus***	β-carotene fucoxanthin PUFA	Able to inhibit LPS-induced inflammatory pathways in human macrophages.	[157]
***Caulerpa cupressoides***	Sulfated polysaccharides	Decrease neutrophils migration. Strongly reduced the carrageenan-induced rat paw edema.	[158]
***Dictyota menstrualis***	Heterofucan	Binds to the surface of leucocytes and inhibits migration of leucocytes to the site of injury. Inhibit the chemical-induced leukocyte migration into the peritoneal cavity.	[159]
***Dictyopteris prolifera*** ***Grateloupia lanceolata*** ***Grateloupia filicina***	Ethanolic extract	Concentration-dependent reduction of LPS-induced prostaglandin E2 production. Suppresses the expression of inducible nitric oxide synthase (iNOS) and cyclooxygenase-2 (COX-2) at the protein level in RAW 264.7 cells. Reduced the release of tumor necrosis factor alpha (TNF-α) and interleukin 6 (IL-6) into the medium.	[160]
***Gracilaria cornea***	Sulfated polysaccharide fraction	Significantly inhibits rat paw edema induced by different inflammatory agents (carrageenan and dextran, histamine and L-arginin). Downregulates interleukin-1β (IL-1β), TNF-α, and COX-2 mRNA and protein levels.	[161]
***Porphyra yezoensis***	Dc-porphyran	Inhibits nitric oxide (NO) production in LPS-stimulated RAW 264.7 cells.	[162]
***Pyropia yezoensis***	Astaxanthin Xanthophyl	Anti-inflammatory action.	[163]
***Lobophora variegata***	Fucans	Inhibits the paw edema, plasma exudation, nitrite content, and leukocyte migration.	[164]
**Red algae**	Carrageenan, Fucoidan, Chondroitin	Lowered the expression of inducible nitric oxide synthase (iNOS). Inhibited the expressions of TNF-α, IL-1β, and interferon-c (IFN-c). Repressed pro-inflammatory cytokines and suppressed the activity of COX-2.	[41,165,166,167]
***Fucus vesiculosus***	Fucoidan	Anti-atopic dermatitis	[103,107]
